# Immune Thrombocytopenic Purpura (ITP) With Ulcerative Colitis (UC)

**DOI:** 10.7759/cureus.72778

**Published:** 2024-10-31

**Authors:** Sohaib K Mohammed, Sanjana Budamagunta, Waleed A Khan, Muneeb Khan, Steven Lippmann

**Affiliations:** 1 Internal Medicine, Countess of Chester Hospital NHS Foundation Trust, Chester, GBR; 2 General Medicine, Deccan College of Medical Sciences, Hyderabad, IND; 3 Acute Internal Medicine, Countess of Chester Hospital NHS Foundation Trust, Chester, GBR; 4 Internal Medicine, Deccan College of Medical Sciences, Hyderabad, IND; 5 Psychiatry, University of Louisville, Louisville, USA

**Keywords:** ibd, immune thrombocytopenia purpura, inflammatory bowel disease, itp, ulcerative colitis (uc)

## Abstract

Inflammatory bowel disease (IBD) includes ulcerative colitis (UC) and Crohn’s disease. A relapsing, immune-modulated illness, it causes inflammation of the small and large intestines.

UC is sometimes associated with extra-intestinal manifestations, including autoimmune cytopenias. Immune thrombocytopenic purpura (ITP) is a rare disorder which may induce an antibody-mediated, isolated reduction in platelet counts. ITP can present with extra-intestinal manifestations; the diagnosis follows the exclusion of other etiologies. Co-occurrences of ITP and UC in patients commonly are at the onset of UC or during illness recurrences. The severity of ITP correlates with the degree of UC pathology. We present a unique case of a 70-year-old presenting ulcerative colitis with immune thrombocytopenic purpura.

## Introduction

Inflammatory bowel disease (IBD) is a chronic, relapsing condition characterized by immune-mediated inflammation of the small and large intestines. Ulcerative colitis (UC) is one such form of IBD, leading to mucosal inflammation starting at the rectum and potentially extending to the colon, with classification based on the extent of involvement. UC is often linked to extra-intestinal manifestations, including hematological abnormalities like autoimmune cytopenias. Among these, immune thrombocytopenic purpura (ITP) is a rare presentation. Although the precise relationship between UC and ITP is not well-defined, antigenic mimicry is hypothesized as a possible mechanism. The co-occurrence of UC and ITP is rare, with studies indicating that ITP affects approximately 0.1%-0.3% of UC patients, suggesting a rare but potential association between these autoimmune disorders [1-3].

## Case presentation

**Figure 1:**
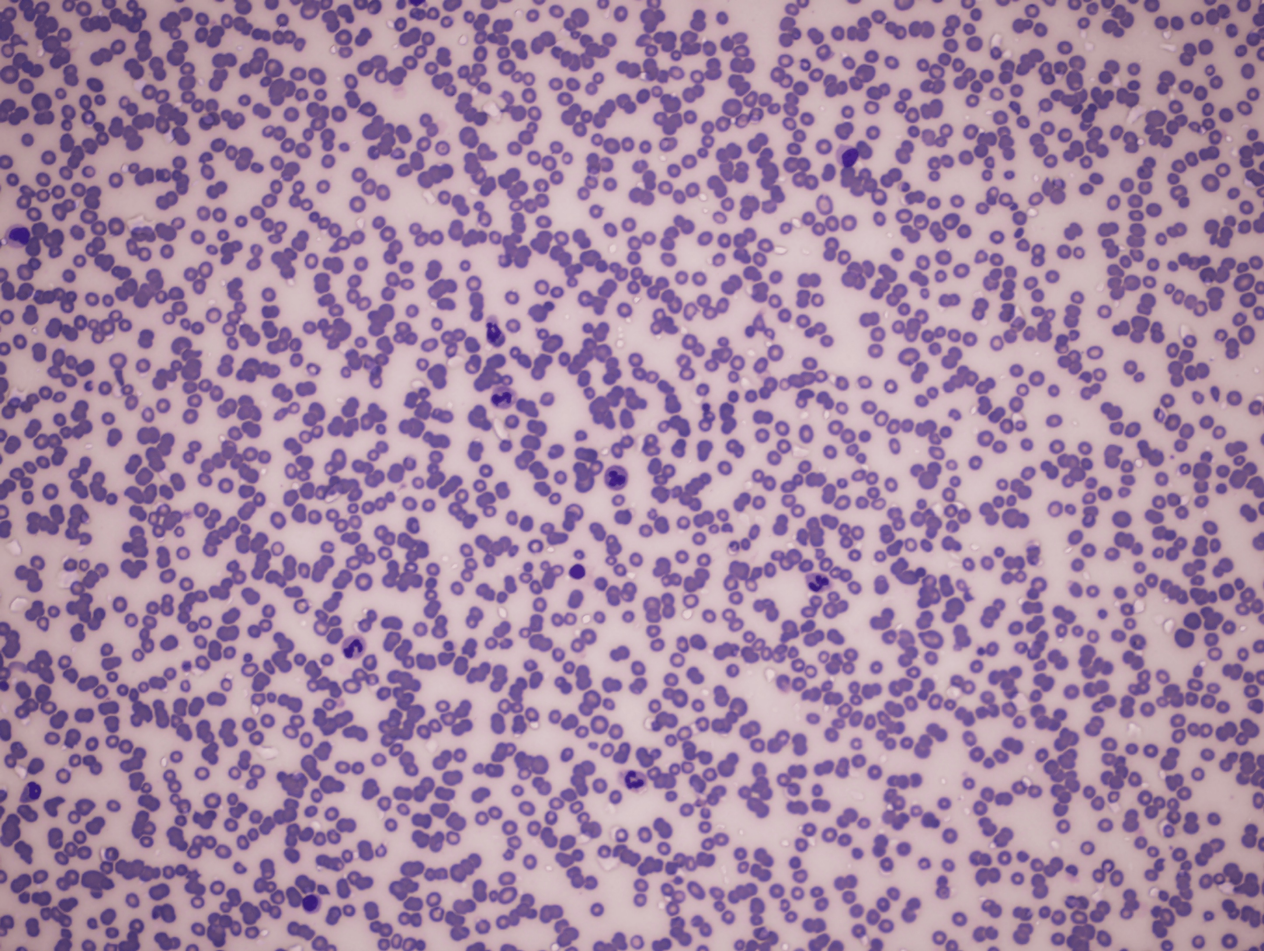
Peripheral smear showing thrombocytopenia

**Figure 2:**
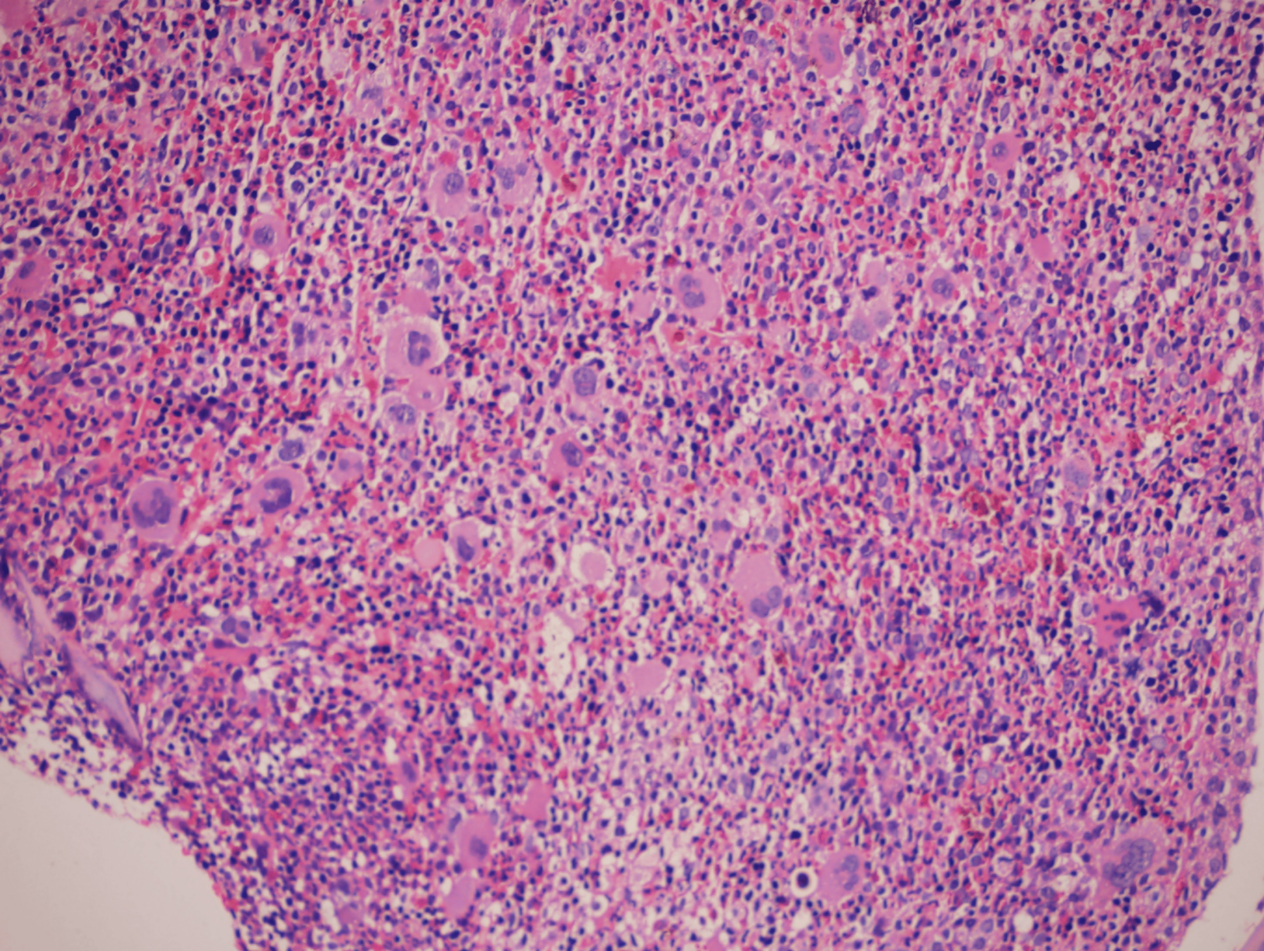
Bone marrow biopsy showing increased megakaryocytes

A 70-year-old female with a history of hypertension and hypothyroidism, presented with complaints of watery, blood-tinged diarrhea. She reported six months of lower abdominal pain, but denied fever, nausea, or vomiting. Vital signs and physical examination were within normal limits, except for lower abdominal tenderness. A hemogram revealed hemoglobin 9.7, WBCs 8,500 (cell/mm 3), packed cell volume (PCV) 30%, mean corpuscular volume (MCV) 87, mean corpuscular hemoglobin (MCH) 28, RBC 3.5 million/mm3, platelet count of 20,000/mm3, ESR 54, serum B12 587 (picograms per milliliter). The chemistries were bilirubin 0.4mg/dl, direct bilirubin 0.1mg/dl, indirect bilirubin 0.3mg/dl, alanine aminotransferase (ALT) 15U/L, aspartate aminotransferase (AST) 32U/L, alkaline phosphatase (ALP) 206U/L, total proteins 8gm/dl, albumin 3gm/dl, globulin 5gm/dl. Thrombocytopenia was noted on peripheral smear (Figure 1) and megakaryocytes were observed at bone marrow biopsy (Figure 2).

A colonoscopy revealed UC, confirmed by biopsy. Oral corticosteroid therapy (1mg/kg/day) was prescribed due to the ITP diagnosis. After hospitalization, platelet counts declined to 15,000/mm3, thus a platelet transfusion was administered. By day eight, her platelet count had increased to 1,00,000. She was discharged stable with a prescription for mesalamine and a tapering course of corticosteroid medicine. 

## Discussion

Ulcerative colitis is an intestinal disorder with inflammation and ulceration of the rectum that may extend to varying lengths of the colonic mucosa. The etiology remains unclear; however, genetic components and/or environmental triggers can disrupt the epithelial barrier of the intestines chronically, leading to gut microbiota intolerance. It thereby provokes a dysregulated immune modulated response. Diagnostic features of UC include bloody diarrhea, tenesmus, and urgency to defecate - sometimes with abdominal cramping. Classification is determined by the extent of mucosal inflammation/ulceration and the length of the colon involved [4].

There are several rare extra-intestinal manifestations, including autoimmune cytopenias and ITP [3].Non-steroidal anti-inflammatory drugs can precipitate disease flare-ups [4,5]. Immune thrombocytopenic purpura is an autoimmune hematologic disorder which may cause an antibody-mediated, isolated reduction in platelet counts. ITP can result in a rapid reduction in platelet numbers by affecting their rate of proliferation in bone marrow and/or following spleen sequestration [6].

The co-occurrence of ITP and UC in patients is most often present at the onset of UC or during a flare-up; thrombocytopenia severity correlates with the degree of colonic pathology. The association is a result of a shared immunological pathway of dysregulation with antigenic mimicry between platelet antigens and those of the intestines. High exposure to bacterial antigens with enhanced intestinal mucosal barrier permeability mediates increased production of antibodies; that causes intestinal mucosal inflammation and platelet antigens that enhance platelet consumption and yields a decreased platelet production rate [7-9]. The antibody-coated platelets are phagocytosed by splenic macrophages. ITP might also be triggered by immune complex-mediated autoantibodies directed against megakaryocytes and/or megakaryocyte precursors, down-regulating platelet turnover [10]. Diagnosed by ruling out other causes of thrombocytopenia [7]. Platelet totals below 50,000/mm3 precipitate bleeding following trauma and when below 20,000/mm3, spontaneous bruising, petechiae, and/or bleeding (e.g., epistaxis) appears. With still lower platelet counts, intracranial/subarachnoid hemorrhage and/or bleeding into the lungs or other organs can occur [6].

The co-occurrence of ITP with UC requires vigilance when evaluating a patient with hematochezia and thrombocytopenia, since the assessment of disease severity (i.e., bloody stools) poses a clinical dilemma with implications on treatment options. ITP management is best directed at its underlying cause [11,12]. With shared immune pathogenesis by UC and ITP, corticosteroid medications are usually effective. In persons with severe thrombocytopenia, the goal is a platelet count that attenuates bleeding. In patients requiring an immediate increase in their platelet numbers, intravenous immunoglobulin infusion is recommended. Platelet transfusions are indicated to control hemorrhages [13,14]. Rituximab is indicated for patients who have failed to respond to other medical interventions. In chronic cases not responsive to medical therapies, splenectomy is considered [15]. People with steroid-refractory ITP and extensive colonic inflammation might improve after surgical colectomy [16]. Thrombocytopenia resulting from destruction of megakaryocytes and megakaryocyte precursors, rather than platelet destruction, may be attenuated by recombinant human thrombopoietin administration [17,18].

## Conclusions

Anyone diagnosed with UC, hematochezia, and a low platelet count ought to be evaluated for the underlying cause of thrombocytopenia. ITP is an important differential diagnosis. Determining disease severity guides treatment planning. This case stands out due to the patient’s advanced age, the presence of comorbid conditions, the severity of thrombocytopenia, the rapid and effective response to treatment, and the specific clinical presentation. These factors collectively highlight the complexity and uniqueness of managing concurrent UC and ITP in an elderly patient with multiple health issues. Such cases underscore the importance of individualized treatment plans and vigilant monitoring to achieve successful outcomes.
